# Glucose Overload Inhibits Glutamatergic Synaptic Transmission: A Novel Role for CREB-Mediated Regulation of Synaptotagmins 2 and 4

**DOI:** 10.3389/fcell.2020.00810

**Published:** 2020-08-19

**Authors:** Cristian Ripoli, Matteo Spinelli, Francesca Natale, Salvatore Fusco, Claudio Grassi

**Affiliations:** ^1^Department of Neuroscience, Università Cattolica del Sacro Cuore, Rome, Italy; ^2^Fondazione Policlinico Universitario A. Gemelli IRCCS, Rome, Italy

**Keywords:** synaptic vesicle release, hippocampus, type 1 diabetes, hyperglycaemia, synaptic proteins, memory loss, metabolism

## Abstract

Glucose metabolism derangement is critically involved in the age-related memory loss but the underlying molecular mechanisms are still poorly understood. In a mouse model of type 1 diabetes we found memory impairment associated with inhibition of the transcription factor CREB and alteration of pre- and post-synaptic protein expression in the hippocampus. Accordingly, glucose excess negatively affected activity-dependent CREB phosphorylation and CREB-mediated mRNA expression of synaptic proteins in hippocampal primary neurons. Specifically, glucose excess inhibited the activity-dependent recruitment of CREB on the regulatory sequences of synaptotagmin (SYT) 2 and 4 promoters and the expression of SYT4 protein. As a result, high glucose affected both the frequency of miniature excitatory postsynaptic currents and NMDA receptor-mediated currents in autaptic hippocampal neuronal cultures. Collectively, our findings highlight novel mechanisms underlying hyperglycaemia-related memory loss, including CREB-dependent downregulation of synaptotagmin expression.

## Introduction

In response to physiological stimuli and environmental conditions, the central nervous system undergoes functional and structural changes (Pascual-Leone et al., 2005). Molecular mechanisms underlying synaptic transmission and plasticity play a pivotal role in the regulation of learning and memory. A plethora of synaptic proteins, including synaptophysin, synapsin family and the SNAP receptor (SNARE) complex regulates the fusion of neurotransmitter vesicles to the synaptic membrane and their release (Brose et al., 2019). Moreover, Ca^2+^ sensor proteins such as synaptotagmins are crucial for Ca^2+^-dependent exocytosis (Fernández-Chacón et al., 2001; Südhof and Rothman, 2009). In glutamatergic synapses, activity-dependent rapid changes in the composition of α-amino-3-hydroxy-5-methyl-4-isoxazolepropionic acid (AMPA) and N-methyl-D-aspartate (NMDA) glutamate receptors at the postsynaptic compartment enhance the amplitude of postsynaptic responses and strengthen the synaptic functions (Zito and Svoboda, 2002).

Importantly, glucose metabolism derangement and type 2 diabetes negatively impact on synaptic plasticity and cognitive functions through multiple mechanisms including oxidative stress, endothelial dysfunctions, microglia activation and neurotrophin depletion (Kullmann et al., 2016; Duffy et al., 2019; Spinelli et al., 2019). However, how glucose excess affects synaptic transmission and neuron-to-neuron communication is still poorly understood. The transcription factor cAMP response element-binding protein (CREB) has been widely investigated as a metabolic sensor and regulator of glucose homeostasis in liver and fat tissue (Altarejos and Montminy, 2011), and as a master switch of Ca^2+^ and neurotrophin-triggered transcriptional programs regulating neuronal differentiation, survival, and plasticity in central and peripheral nervous systems (Riccio et al., 1999; Finkbeiner, 2000, Mantamadiotis et al., 2002). Furthermore, brain plasticity and high-order cognitive functions are as well influenced by nutrient cues and energy metabolism through CREB-dependent expression of genes involved in adult neurogenesis, chromatin silencing and neuronal metabolism (Fusco et al., 2012, 2016). Here we demonstrated that glucose overload inhibits CREB activity in hippocampal neurons and alters the expression of genes encoding pre- and post-synaptic proteins. High glucose concentration also impairs the spontaneous vesicle release and both the amplitude and the kinetics of NMDA receptor-mediated currents at the post-synaptic level. Finally, we identified synaptotagmin 2 and 4 (SYT2 and SYT4, respectively) as novel glucose-responsive CREB target genes involved in hyperglicaemia-dependent impairment of synaptic function.

## Materials and Methods

### Ethics Statement

The animal study was reviewed and approved by the Ethics Committee of Università Cattolica del Sacro Cuore and were fully compliant with Italian (Ministry of Health guidelines, Legislative Decree No. 116/1992) and European Union (Directive No. 86/609/EEC) legislations on animal research.

### Animals

Male C57BL/6 mice (30 days-old), derived from the Animal Facility of Università Cattolica del Sacro Cuore, were used and randomly assigned to two treatments: (i) intraperitoneal injection of saline (CTR, control) and (ii) intraperitoneal injection of streptozotocin (STZ). Mice were housed in groups (3–5 animals per cage) and they were daily monitored. To induce hyperglycaemia, mice were intraperitoneally injected with 50 mg/Kg of streptozotocin (Sigma-Aldrich) for five consecutive days. The drug was freshly prepared in Na^+^ citrate solution buffered to 4.5 pH. Before each streptozotocin injection, animals were fasted for 6 h. After each injection, mice were supplied with 10% sucrose water to avoid sudden hypoglycaemia post injection. Mice were tested for sufficient levels of hyperglycaemia at 2 weeks after last injection (defined as time 0) and only mice with plasma glucose levels higher than 300 mg/dL were used for experiments. Blood sugar dosages and behavioral analyses were performed at both 1 and 3 weeks from time 0. Molecular analyses were performed on whole hippocampi collected 3 weeks from time 0 on different cohorts of mice. The animals were housed under a 12-h light-dark cycle at room temperature (RT: 19–22 °C) and received both food and water *ad libitum.*

### Culture of Primary Neurons

Primary cultures of hippocampal neurons were obtained from E18 C57BL/6 mice embryos according to standard procedures. Briefly, hippocampi were dissected and incubated for 10 min at 37 °C in PBS containing 0.025% trypsin/0.01% EDTA (Biochrom AG). The tissue was then mechanically dissociated at room temperature (23–25 °C) using a fire-polished Pasteur pipette and the cell suspension was harvested and centrifuged at 100 × *g* for 8 min. The pellet was suspended in 88.8% (vol/vol) minimum essential medium (Biochrom), 5% FBS, 5% (vol/vol) horse serum, 1% glutamine (2 mM), 0.2% gentamicin (0.1 mg/mL) and glucose (25 mM). At 24 h after plating (1st day in vitro, DIV1), the culture medium was replaced with a medium containing 97.3% (vol/vol) neurobasal medium (Invitrogen), 2% (vol/vol) B-27 (Invitrogen), 0.5% glutamine (2 mM), and 0.2% gentamicin (0.1 mg/mL). After 72 h (DIV4), the culture medium was replaced with a similar medium lacking glutamine and supplemented with 2 μM cytosineβ-D-arabinofuranoside to inhibit glial cell proliferation. Autaptic hippocampal neurons were prepared as previously described (Attar et al., 2012; Ripoli et al., 2013). In brief, cortical astrocytes from P0–P2 brains of C57BL/6 mice were plated onto agarose-coated glass coverslips on which microislands where astrocytes could be grown were created by spraying a mixture of poly-D-lysine and collagen (both from Sigma). After 4 days, the medium (consisting of DMEM supplemented with 10% fetal bovine serum and antibiotics) was conditioned replacing half the medium volume with neuronal medium (Neurobasal medium, 2% B-27, 0.5% glutamine, and 1% penicillin-streptomycin-neomycin antibiotic mixture). Hippocampal neurons from P0 to P2 C57BL/6 were plated onto glial microislands at low density (25,000/cm^2^) to obtain a ratio of one neuron per island. Both cultures (hippocampal neurons and autaptic hippocampal neurons) were maintained at 37°C in a humidified atmosphere of 5% CO_2_ until experimental procedures. Every 3 days glucose levels in neuronal media were analyzed with glucometer. At DIV7 and DIV11 half the medium volume was replaced with fresh medium containing 25 mM (HG) or 0–5.5 mM (NG) in order to gradually decrease the level of glucose in NG samples and to obtain the experimental glucose concentration at DIV11. Molecular and electrophysiological experiments were performed at DIV14, after 3 days of HG (25 mM) and NG (5.5 mM). For molecular analyses, hippocampal neurons were stimulated with either 20 mM potassium chloride (Sigma Aldrich) or 10 μM forskolin (Sigma Aldrich). These compounds were applied for 30 min to investigate CREB phosphorylation and for 6 h to study gene expression modifications.

### Behavioral Experiments

Behavioral tests were carried out from 9 a.m. to 4 p.m. and data were analyzed in blind using an automated video tracking system (Any-Maze^TM^). Recognition memory was evaluated by novel object recognition (NOR) test. On first day, animals were familiarized for 10 min to the test arena (45 cm × 45 cm). On second day (training session), they were allowed to explore two identical objects placed symmetrically in the arena for 10 min. Mice exhibiting a total exploration time lower than 30 s or exploring one of two identical objects for more than 60% of the total exploration time during training session were excluded from the test. On third day (test session), a new object replaced one of the old objects. Animals were allowed to explore for 10 min and preference index, calculated as the ratio between time spent exploring the novel object and time spent exploring both objects, was used to measure recognition memory. To exclude place preference in the test session, the position of novel object was alternated when testing the different animals. All objects and the box were cleaned with 70% ethanol at the end of each test.

### Western Blotting

Tissues (hippocampi) or cells (hippocampal neurons) were lysed in ice-cold lysis buffer (NaCl 150 mM, Tris–HCl 50 mM pH 7.4, EDTA 2 mM) containing 1% Triton X-100, 0.1% SDS, 1 × protease inhibitor cocktail (Sigma-Aldrich), 1 mM sodium orthovanadate (Sigma-Aldrich) and 1 mM sodium fluoride (Sigma-Aldrich). Cells were incubated for 10 min on ice with occasional vortexing and spun down at 22,000 × g, 4°C. Supernatant was quantified for protein content (DC Protein Assay; Bio-Rad). Equal amounts of protein were diluted in Laemmli buffer, boiled and resolved by SDS-PAGE. The primary antibodies (available in Supplementary Table S1) were incubated overnight and revealed with HRP-conjugated secondary antibodies (Cell Signaling Technology Inc., Danvers, MA) and chemiluminescent substrates (Cyanagen). Band density was assessed by using UVItec Cambridge Alliance (Cambridge, United Kingdom). Protein expression levels were quantified by calculating the band intensity ratio of the target protein and actin (loading control) in each lane. Phosphorylation level of target proteins was quantified by calculating the band intensity ratio of phospho-target protein, target protein and actin (loading control) in each lane. In each bar graph, the mean value of controls was set to 1 and the expression or phosphorylation levels of target protein were shown as fold changes compared to the control (relative units). Images shown were cropped for presentation with no manipulations.

### Real-Time PCR

Quantitative Real-Time PCR (qRT-PCR) amplifications were performed using SYBR GREEN qPCR Master Mix (Fisher Molecular Biology) on AB7500 instrument (Life Technologies) according to the manufacturer’s instructions. The thermal cycling profile featured a pre-incubation step of 94°C for 10 min, followed by 40 cycles of denaturation (94°C, 15 s), annealing (55°C, 30 s), and elongation (72°C, 20 s). Melting curves were subsequently generated (94°C for 15 s, 50°C for 30 s, slow heating to 94°C in increments of 0.5°C).

Melting-curve analyses confirmed that only single products had been amplified. The primer sequences are shown in Supplementary Table S2. All data were normalized by reference to the amplification levels of the Gapdh gene; a reference dye was included in the SYBR master mix. RNA of all samples was analyzed in triplicate. The thresholds calculated by the software were used to determine specific mRNA expression levels using the cycle-at-threshold (Ct) method, and all results are expressed as fold changes (compared to control) for each transcript, employing the 2−ΔΔCt approach.

### Immunocytochemistry

Hippocampal neurons were fixed in PBS solution (4% PFA, pH 7.4; Sigma-Aldrich) for 15 min at RT. Neurons were then permeabilized with 0.2% Triton X-100 (Sigma-Aldrich) for 15 min, blocked for 60 min in 5% NGS, and then incubated overnight at 4°C with anti-MAP2 (HM-2 clone, 1:400, Sigma-Aldrich). Cells were subsequently incubated for 90 min at RT with secondary antibody (Alexa-Fluor Donkey Anti-Mouse 1:1000). Finally, nuclei were counterstained with 4′, 6- diamidino-2-phenylindole (DAPI, 0.5 μg per mL for 10 min; Thermo Fisher), and cells were coverslipped with ProLong Gold anti-fade reagent (Thermo Fisher). Images of 1024 × 1024 pixels were obtained with an A1 MP, Nikon confocal microscope (Tokyo, Japan) equipped with 20× and 40× magnification objectives (numerical aperture 1.4), plus additional magnification.

### Electrophysiology in Autaptic Microcultures

All electrophysiological recordings were performed using whole-cell patch clamp. Recordings were obtained with an Axopatch 200B amplifier (Molecular Devices), and stimulation and data acquisition were performed with the Digidata 1200 series interface and pCLAMP 11 software (Molecular Devices). Basal synaptic transmission was studied from 14 to 21 DIV using the patch-clamp technique in the whole-cell configuration as previously described (Ripoli et al., 2014). Cells were approached under DIC with 3–5 MΩ pipettes pulled from borosilicate glass (Warner Instruments, Inc) using a vertical Narishige PC-10 puller (Japan) and filled with an internal solution containing (in mM): 146 K-gluconate, 18 HEPES, 1 EGTA, 4.6 MgCl_2_, 4 NaATP, 0.3 Na_2_GTP, 15 creatine phosphate, and 5 U/ml phosphocreatine kinase. External Tyrode’s solution containing the following (in mM): 140 NaCl, 2 KCl, 10 HEPES, 10 glucose, 4 MgCl_2_, and 4 CaCl_2_, pH 7.4, 312 mOsm. NMDA receptor-mediated currents were evoked using Mg-free Tyrode’s solution containing 10 mM of the AMPA receptor blocker NBQX (Tocris Bioscience). Neurons were maintained at −70 mV holding potentials, and EPSCs were elicited with stimuli mimicking action potentials (2 ms at 0 mV) delivered every 10 s or 20 s. The paired-pulse ratio consisted of the ratio of the amplitude of the second EPSC to that of the first recorded at 50 ms intervals (Fattorini et al., 2019). To obtain the AMPA/NMDA ratio, evoked responses were recorded successively from the same cell. The amplitude and frequency of miniature excitatory postsynaptic currents (mEPSCs) were evaluated in 60 s recordings. The decay time was estimated by fitting a single exponential to the 10–90% decay-phase. We monitored the access resistance and membrane capacity before and at the end of the experiments to ensure recording stability and the health of studied cells. Whole-cell recordings were performed 5–15 min after the culture medium replacement with external Tyrode’s solution. The culture plates were changed every half-hour. All experiments were performed at RT.

### Chromatin Immunoprecipitation

Chromatin immunoprecipitation (ChIP) assays were performed as previously described (Fusco et al., 2019). Neurons were resuspended in 200 μl lysis buffer containing 1% SDS, 50 mM Tris–HCl pH 8.0, and 10 mM EDTA and sonicated on ice with six 10-s pulses with a 20-s interpulse interval. Sample debris was removed by centrifugation and supernatants were precleared with protein-G Sepharose 4B beads (Sigma-Aldrich) for 1 h at 4°C. 2 μg of anti-CREB or control IgG were added overnight at 4°C. Immune complexes were collected by incubation with protein-G Sepharose 4B beads for 2 h at 4°C. After seven sequential washes, immune complexes were eluted from beads by vortexing in elution buffer (1% SDS and NaHCO_3_ 0.1 M; pH 8.0). NaCl was added (final concentration 0.33 M), and cross-linking was reversed by incubation overnight at 65 °C. DNA fragments were purified by using the PCR DNA fragments purification kit (Geneaid). The primer sequences are shown in Supplementary Table S2.

PCR conditions and cycle numbers were determined empirically and each PCR reaction was performed in triplicate. Data are expressed as percentage of input calculated by the “Adjusted input value” method according to the manufacturer’s instructions (ThermoFisher Scientific ChIP Analysis). To determine the Adjusted input the Ct value of input was subtracted by 6.644 (i.e., log2 of 100). Next, the percent input of samples was estimated using the formula: 100^∗^2^(Adjusted input – Ct(ChIP). The percent input of IgG samples was calculated using the formula 100^∗^2^(Adjusted input – Ct(IgG).

### Statistical Analysis

Sample sizes were chosen with adequate power (0.8) according to results of prior pilot data sets or studies, including our own, which used similar methods or paradigms. Sample estimation and statistical analyses were performed using SigmaPlot 12 software. Data were first tested for equal variance and normality (Shapiro-Wilk test) and the appropriate statistical tests were chosen. The statistical tests used (i.e., Student’s *t*-test, two-way ANOVA) are indicated in the main text and in the corresponding figure legends for each experiment. N numbers are reported in the figure legends. Degrees of freedom are n–1 for each condition in both unpaired *t*-test and ANOVA tests. *Post-hoc* multiple comparisons were performed with Bonferroni correction. All statistical tests were two-tailed and the level of significance was set at 0.05. Results are shown as mean ± SEM.

## Results

### Hyperglicemia Reduces the Expression of Pre- and Post-synaptic Proteins in the Hippocampus

**FIGURE 1:**
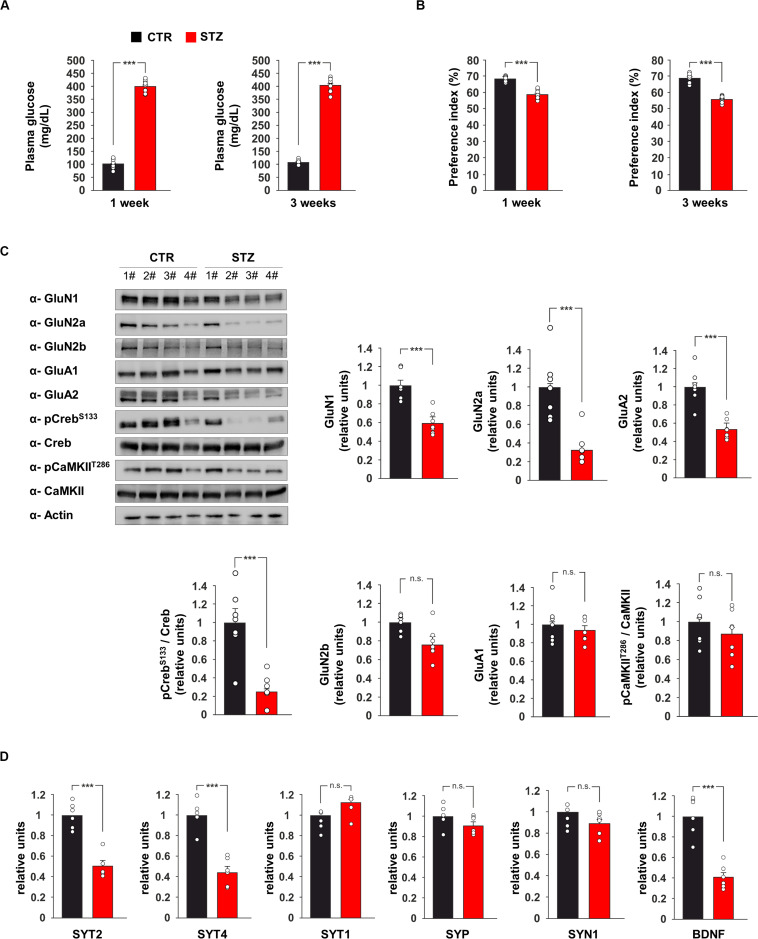
Hyperglicaemia affects the expression of pre- and post-synaptic proteins in the hippocampus. **(A)** Plasma levels of glucose in C57BL/6 mice i.p. injected with saline (CTR) or streptozotocin (STZ, 50 mg/Kg/day for 5 days). Glycaemia levels were detected after 1 or 3 weeks from the onset of hyperglycaemia in STZ mice (*n* = 9 mice per group; statistics by unpaired Student’s *t*-test). **(B)** Preference for the novel object in the NOR paradigm after 1 or 3 weeks from the onset of hyperglycaemia in CTR and STZ mice (*n* = 9 mice; statistics by unpaired Student’s *t*-test). **(C)** Immunoblot analysis and bar graphs showing the expression of GluN1, GluN2a, GluN2b, GluA1, GluA2 and the phosphorylation of both Creb on serine 133 (Creb^S133^) and CaMKII on threonine 286 (CaMKII^T286^) in the hippocampus of CTR and STZ mice (*n* = 7 mice; statistics by unpaired Student’s *t*-test). **(D)** mRNA expression of synaptotagmin 1, 2 and 4 (SYT1, SYT2, and SYT4, respectively), synaptophysin (SYP), synapsin 1 (SYN1) and BDNF in the hippocampus of CTR and STZ mice (*n* = 6 mice; statistics by unpaired Student’s *t*-test). Real Time analysis was performed in triplicate. Gene expression was normalized to Gapdh. Data are expressed as mean ± SEM. *p < 0.05; ***p < 0.001; n.s. not significant.

Glucose metabolism dysregulation has been reported to affect synaptic function (Zhong et al., 2019). Previous studies demonstrated alterations of hippocampus-dependent-learning and memory in experimental models of hyperglycaemia (Gispen and Biessels, 2000) but the underlying molecular mechanisms remain still poorly understood. To investigate the effects of glucose excess on the expression of pre- and post-synaptic proteins in the hippocampus, we set up an *in vivo* model of streptozotocin (STZ)-induced hyperglycaemia. First, we evaluated glucose plasma levels and hippocampus-dependent cognitive function one and 3 weeks after the onset of hyperglicaemia. As expected, multiple STZ injections induced elevated values of fasting glycaemia and this alteration persisted after 3 weeks (400.44 ± 8.29 mg dL^–1^ vs 102.77 ± 6.06 mg dL^–1^, *p* = 1.11 × 10^–15^ after 1 week; 406.22 ± 9.38 mg dL^–1^ vs 107.00 ± 3.28 mg dL^–1^, *p* = 6.09 × 10^–16^ after 3 weeks; Figure 1A). More importantly, STZ mice already showed lower preference index than controls after the first week of high glucose levels, and their performances in novel object recognition (NOR) task even got worse after 3 weeks of hyperglicaemia (after 1 week: preference index 59.0 ± 0.9% vs 68.5 ± 0.5%, *p* = 7.05 × 10^–8^, exploration time toward novel object/old object 40.7 ± 6.7 s / 29.5 ± 8.2 s vs 42.2 ± 6.5 s / 19.4 ± 4.2 s; after 3 weeks: preference index 56.0 ± 0.7% vs 69.0 ± 1.1%, *p* = 1.23 × 10^–8^, exploration time toward novel object/old object 41.1 ± 4.4 s / 32.3 ± 4.6 s vs 47.1 ± 4.9 s / 21.1 ± 3.2 s; Figure 1B).

We also investigated the expression of glutamate receptor subunits and the activity-dependent phosphorylation of neuroplasticity proteins CaMKIIα and CREB in the hippocampus of hyperglicaemic mice. The immunoblot analysis of hippocampal lysates from STZ mice revealed lower expression of NMDA receptor subunits GluN1 and GluN2a and AMPA receptor subunit GluA2 compared to controls (−41 ± 5, −67 ± 8, and −47 ± 4%, respectively, *p* < 0.001 for all proteins; Figure 1C). In addition, STZ treatment reduced the activatory phosphorylation of transcription factor CREB on serine 133 (CREB^Ser133^) (−75 ± 7%, *p* = 0.0004; Figure 1C). Instead, no significant changes were observed for the expression of GluN2b and GluA1 and the activatory phosphorylation of CaMKIIα on threonine 286 (Figure 1C). CREB-mediated transcription of presynaptic proteins has been demonstrated to promote synaptic enhancement and memory (Wagatsuma et al., 2006). Therefore, we analyzed the mRNA expression of several synaptic transmission-associated proteins in the hippocampus of STZ mice. We found lower expression of synaptotagmin 2 and 4 (SYT2 and SYT4, respectively) in hyperglycaemic mice (−49 ± 5%, *p* = 3.34 × 10^–5^ and −56 ± 6%, *p* = 3.96 × 10^–5^, respectively; Figure 1D), whereas we did not detect any significant changes of synaptotagmin 1 (SYT1), synaptophysin (SYP) and synapsin 1 (SYN1). We also found a significant decrease of Bdnf expression in the hippocampus of STZ mice compared to controls (−59 ± 5%, *p* = 7.12 × 10^–5^; Figure 1D). Collectively, *in vivo* data demonstrated that STZ-induced hyperglycaemia reduced CREB activation and the expression of hippocampal pre- and post-synaptic proteins involved in synaptic function and memory.

### Glucose Excess Inhibits CREB-Dependent Gene Expression

**FIGURE 2:**
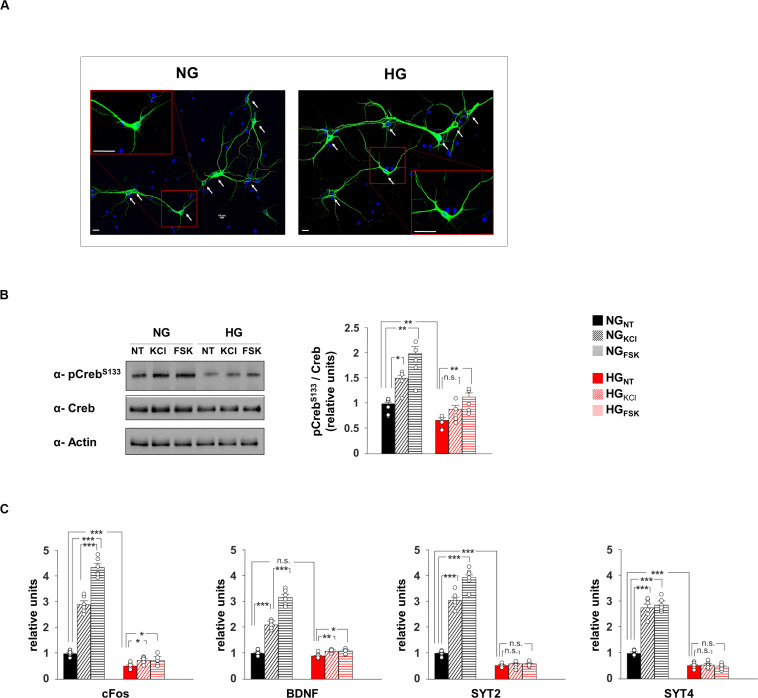
Glucose excess impairs both CREB phosphorylation and expression of plasticity-related genes in neurons. **(A)** Representative images of hippocampal neurons cultivated in media containing normal (NG, 1.0 g/L) or high glucose (HG, 4.5 g/L) levels. Scale bar: 50 μM. **(B)** Immunoblot analyses and bar graphs showing CrebS133 phosphorylation in NG or HG neurons treated with vehicle (NT), 20 mM potassium chloride (KCl) or 10 μM forskolin (Fsk). The experiment was repeated five times (statistics by two-way ANOVA and Bonferroni *post hoc*). **(C)** mRNA expression of cFos, BDNF, SYT2, and SYT4 in NH and HG neurons. Real Time analysis was performed in triplicate. Gene expression was normalized to Gapdh. The experiment was repeated six times using independent RNA samples (statistics by two-way ANOVA and Bonferroni post hoc). Data are expressed as mean ± SEM. *p < 0.05; **p < 0.01; ***p < 0.001; n.s. not significant.

To deeply investigate the effect of hyperglycaemia on CREB transcriptional activity, we set up an *in vitro* model of hippocampal primary neurons cultivated in media containing either normal (NG) or high glucose (HG) concentrations (1 g/L or 4.5 g/L, respectively; Figure 2A). In neurons, synaptic activity enhances the intracellular concentration of Ca^2+^ and cyclic adenosine monophosphate (cAMP), both leading to phosphorylation and activation of CREB (Deisseroth et al., 1996). Accordingly, compounds inducing intracellular increase of either Ca^2+^ (20 mM KCl) or cAMP (10 μM forskolin [Fsk]) induced CREB^Ser133^ phosphorylation in neurons exposed to normal glucose levels (*F*_2.71_ = 16.82, +49 ± 12% NG_KCl_ vs NG_NT_, *p* = 0.011; +99 ± 18% NG_F__sk_ vs NG_NT_, *p* = 0.004, Figure 2B). Conversely, HG significantly reduced the basal phosphorylation levels of CREB and inhibited its activation upon KCl stimulation (−34 ± 6% HG_NT_ vs NG_NT_, *p* = 0.009; +33 ± 9% HG_KCl_ vs HG_NT_, *p* = 0.088, Figure 2B). We also analyzed the mRNA expression of both CREB target genes and synaptic proteins that we found downregulated in the hippocampus of STZ mice. In standard conditions, both KCl and Fsk largely enhanced the transcription of CREB targets c-Fos and Bdnf, whereas this enhancement was cut down by HG treatment (*F*_2.60_ = 270.15 for cFos, +189 ± 14% NG_KCl_ vs NG_NT_, *p* = 8.39 × 10^–6^; +334 ± 16% NG_Fsk_ vs NG_NT_, *p* = 5.86 × 10^–7^; +48 ± 5% HG_KCl_ vs HG_NT_, *p* = 0.012; +48 ± 8% HG_Fsk_ vs HG_NT_, *p* = 0.038; *F*_2.60_ = 191.32 for Bdnf, +109 ± 7% NG_KCl_ vs NG_NT_, *p* = 1.06 × 10^–7^; +215 ± 13% NG_Fsk_ vs NG_NT_, *p* = 6.85 × 10^–7^; +20 ± 2% HG_KCl_ vs HG_NT_, *p* = 0.006; +19 ± 4% HG_Fsk_ vs HG_NT_, *p* = 0.015; Figure 2C). Moreover, molecules activating CREB enhanced the expression of both SYT2 and SYT4 genes. More importantly, glucose excess lowered the transcription of synaptotagmins and abolished their CREB activity-related upregulation reproducing *in vitro* the molecular changes observed *in vivo* (*F*_2.60_ = 286.79 for SYT2, +203 ± 14% NG_KCl_ vs NG_NT_, *p* = 5.36 × 10^–6^; +292 ± 16% NG_Fsk_ vs NG_NT_, *p* = 1.57 × 10^–6^; −49 ± 3% HG_NT_ vs NG_NT_, *p* = 6.82 × 10^–6^; HG_KCl_ vs HG_NT_, *p* = 0.955; HG_Fsk_ vs HG_NT_, *p* = 0.254; *F*_2.60_ = 188.26 for SYT4, +176 ± 16% NG_KCl_ vs NG_NT_, *p* = 2.24 × 10^–5^; +187 ± 11% NG_Fsk_ vs NG_NT_, *p* = 5.37 × 10^–7^; −49 ± 5% HG_NT_ vs NG_NT_, *p* = 1.72 × 10^–5^; HG_KCl_ vs HG_NT_, *p* = 0.711; HG_Fsk_ vs HG_NT_, *p* = 0.511; Figure 2C). Our data indicate that HG negatively impacts on CREB phosphorylation and its transcriptional activity in hippocampal neurons, correlating with the impairment of synaptic protein expression.

### HG Inhibited the Recruitment of CREB on Both SYT2 and SYT4 Promoters

**FIGURE 3:**
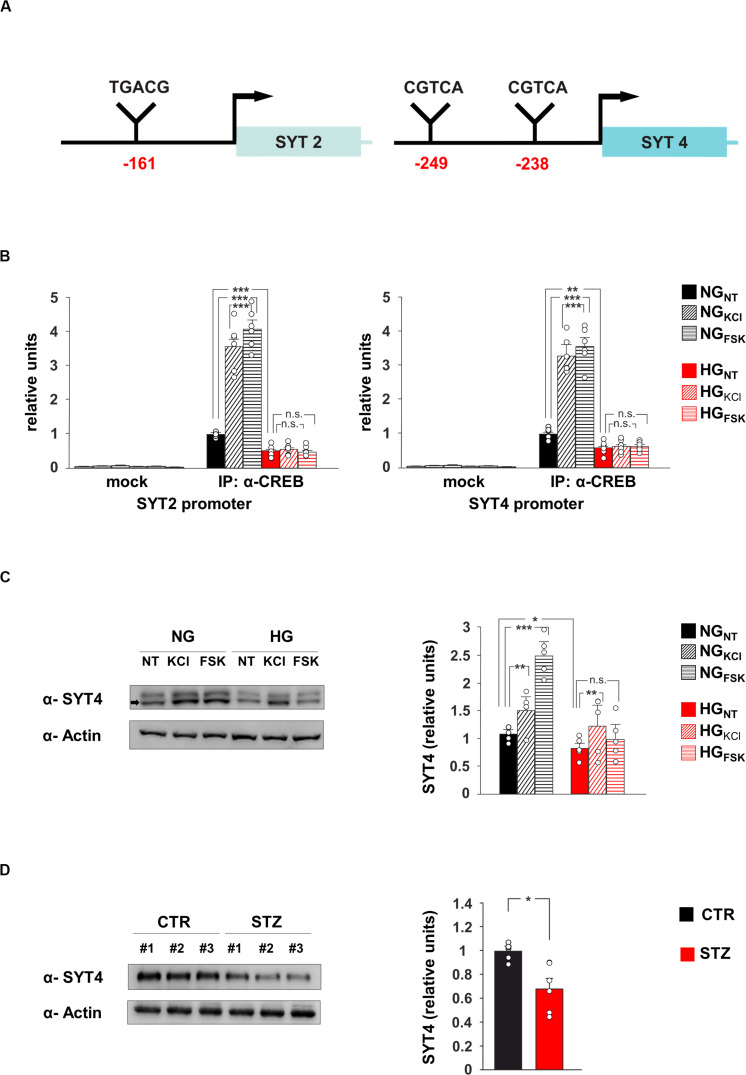
CREB regulates SYT4 in a glucose-dependent fashion. **(A)** Schematic representation of putative CRE regions identified on the promoters of SYT2 (–161 bp from transcription start site) and SYT4 (–249 and –238 bp upstream the starting codon) genes. **(B)** ChIP analysis of both non-specific IgG (mock) and CREB binding to the SYT2 and SYT4 promoters in NG and HG neurons stimulated with vehicle (NT), 20 mM potassium chloride (KCl) or 10 μM forskolin (Fsk). Real Time analysis was performed in triplicate. Experiments were repeated six times using independent DNA samples (statistics by two-way ANOVA and Bonferroni post hoc). **(C)** Immunoblot analysis and bar graphs showing the expression of SYT4 in NG or HG neurons treated with vehicle, KCl or Fsk. The experiment was repeated five times (statistics by two-way ANOVA and Bonferroni post hoc). **(D)** Immunoblot analysis and bar graphs showing the expression of SYT4 in the hippocampus of CTR and STZ mice (*n* = 6 mice; statistics by unpaired Student’s *t*-test). Data are expressed as mean ± SEM. *p < 0.05; **p < 0.01; ***p < 0.001; n.s. not significant.

The transcription factor CREB modulates synaptic activity by modifying its binding on the promoters of neuronal genes and regulating their expression (West and Greenberg, 2011). Our data suggested that SYT2 and SYT4 might represent novel molecular targets of CREB and be involved in the HG-related alteration of synaptic function. To verify whether the HG-dependent inhibition of CREB activity was implicated in the changes of SYT2 and SYT4 expression, we first analyzed the regulatory sequences of these genes. The bioinformatics analysis revealed the presence of several putative cAMP Responsive Elements (CRE) on the regulatory sequences of both SYT2 and SYT4 (Figure 3A). Chromatin immunoprecipitation experiments from hippocampal neurons showed that CREB binds the same genomic region in a fashion inducible by KCl and Fsk (*F*_2.60_ = 102.19 for SYT2, +256 ± 31% NG_KCl_ vs NG_NT_, *p* = 2.87 × 10^–4^; +308 ± 25% NG_Fsk_ vs NG_NT_, *p* = 4.63 × 10^–5^; *F*_2.60_ = 99.53 for SYT4, +229 ± 23% NG_KCl_ vs NG_NT_, *p* = 4.5 × 10^–5^; +257 ± 25% NG_Fsk_ vs NG_NT_, *p* = 3.47 × 10^–5^; Figure 3B). Moreover, glucose excess affected the recruitment of transcription factor on the promoters of SYT2 and SYT4 in both basal and inducible conditions (*F*_2.60_ = 102.19 for SYT2, −51 ± 8% HG_NT_ vs NG_NT_, *p* = 3.28 × 10^–4^; HG_KCl_ vs HG_NT_, *p* = 0.422; HG_Fsk_ vs HG_NT_, *p* = 0.732; *F*_2.60_ = 99.53 for SYT4, −40 ± 8% HG_NT_ vs NG_NT_, *p* = 3.61 × 10^–3^; HG_KCl_ vs HG_NT_, *p* = 0.740; HG_Fsk_ vs HG_NT_, *p* = 0.620; Figure 3B). Accordingly, CREB-activating stimuli induced SYT4 expression in NG-treated hippocampal neurons, whereas HG decreased SYT4 at protein level and inhibited its Fsk-dependent upregulation (*F*_2.71_ = 49.66, +43 ± 4% NG_KCl_ vs NG_NT_, *p* = 0.0019; +136 ± 15% NG_Fsk_ vs NG_NT_, *p* = 2.29 × 10^–5^; −22 ± 2% HG_NT_ vs NG_NT_, *p* = 0.018; +21 ± 11% HG_Fsk_ vs HG_NT_, *p* = 0.089; Figure 3C). Moreover, SYT4 expression was significantly reduced in the hippocampi of hyperglycaemic mice (−32 ± 9%, *p* = 0.0102; Figure 3D). Collectively, our findings identify SYT2 and SYT4 as novel activity-dependent targets of CREB and indicate the CREB-dependent downregulation of vesicle release as a potential mechanism leading HG-dependent impairment of glutamatergic synaptic transmission.

### HG Alters the Basal Glutamatergic Synaptic Transmission in Autaptic Hippocampal Neurons

**FIGURE 4:**
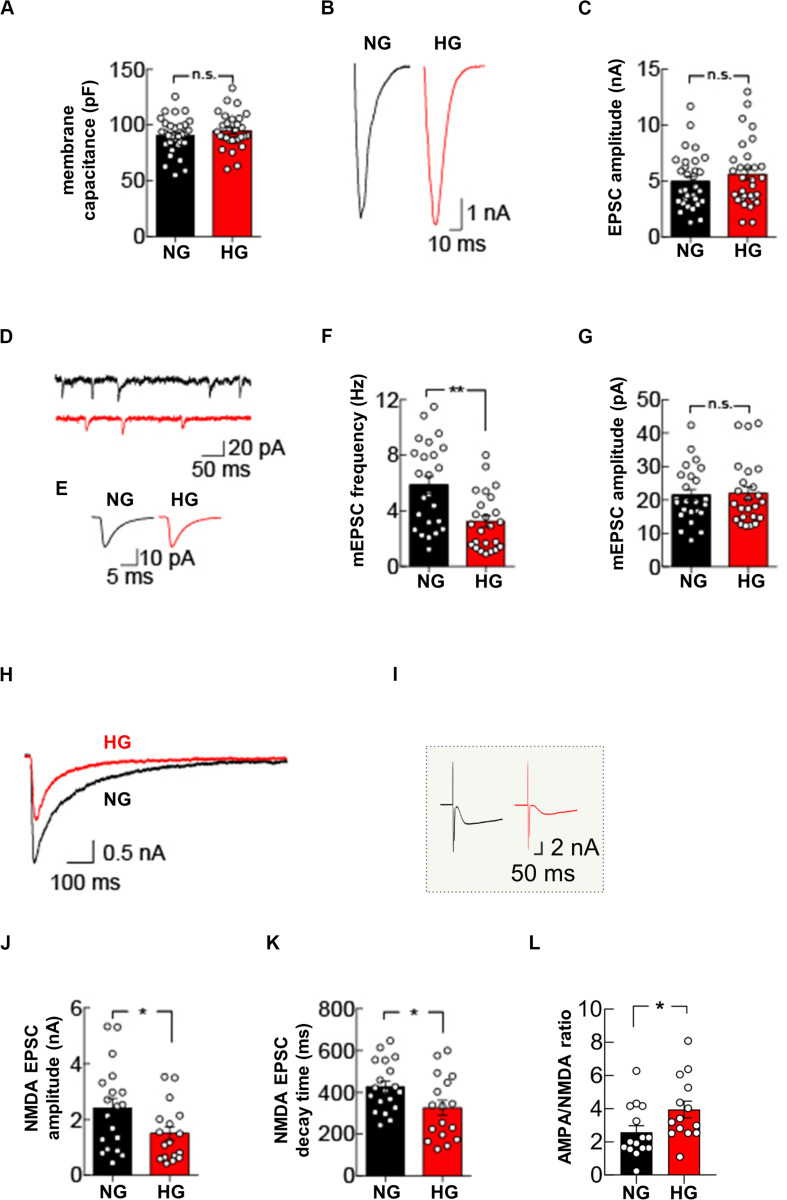
High glucose treatment impairs mEPSC frequency and NMDA receptor-mediated currents. **(A)** Quantification of membrane capacitance recorded in autaptic microcultures grown in NG (*n* = 31) or HG conditions (*n* = 30). **(B)** Representative traces of EPSC evoked by stimuli mimicking single action potentials recorded in NG- and HG-treated autaptic neurons. **(C)** Summary graphs of EPSC amplitudes recorded from autaptic neurons grown in either NG (*n* = 31) or HG (*n* = 31). **(D)** Example traces showing spontaneous mEPSCs from NG- and HG-treated autaptic neurons. **(E)** Representative mEPSC averaged traces. Summary graphs of mEPSC frequency (*n* = 24 per group) **(F)** and mEPSC amplitudes (*n* = 24 per group) **(G)**. **(H)** Representative traces of NMDA receptor-mediated currents evoked by stimuli mimicking single action potentials and recorded in NG- and HG-treated autaptic neurons. (**I**) Inset: raw traces of H. Summary graphs of NMDA receptor-mediated currents amplitude **(J)** and decay time **(K)** from autaptic neurons grown in NG (*n* = 19) and in HG (*n* = 17). **(L)** Summary graphs of the AMPA:NMDA ratio recorded from autaptic neurons grown in either NG (*n* = 15) or HG (*n* = 14). Data are expressed as mean ± SEM. *p < 0.05; **p < 0.001; n.s. not significant. Statistic by Mann–Whitney U-test, mEPSC amplitudes were analyzed with the Kolmogorov-Smirnov test.

Our molecular data demonstrate that glucose excess can alter the expression of pre- and post-synaptic proteins in hippocampal neurons. To evaluate the functional role of glucose dyshomeostasis on glutamatergic synaptic transmission, we performed patch-clamp experiments in autaptic hippocampal neuronal cultures grown in NG or HG conditions. First, we measured the membrane capacitance of autaptic hippocampal neurons that was unchanged by HG treatment (90.5 ± 2.9 pF in NG condition *vs* 94.8 ± 3.0 pF in HG condition, *p* = 0.3994; Figure 4A).

Glucose overload did not affect evoked basal synaptic transmission, measured as the amplitude of excitatory postsynaptic currents (EPSCs) elicited by stimuli mimicking action potentials (5.0 ± 0.4 nA in NG condition *vs* 5.6 ± 0.5 nA in HG condition, *p* = 0.4155; Figures 4B,C). Analysis of mEPSCs in autapses exposed to HG revealed a significant decrease in the mEPSC frequency (5.9 ± 0.6 Hz in NG condition vs 3.2 ± 0.4 Hz in HG condition, *p* = 0.0011; Figures 4D–F) whereas the mean amplitude and the kinetics of mEPSCs (i.e., rise and decay time constants) were unaffected (mEPSC amplitude: 21.5 ± 1.7 pA in NG condition vs 22.0 ± 2.0 pA in HG condition, *p* = 0.8928, Figures 4D,E,G; mEPSC rise time: 1.1 ± 0.1 ms in NG condition vs 1.0 ± 0.1 ms in HG condition, *p* = 0.5279; mEPSC decay time 3.5 ± 0.2 ms in NG condition vs 3.7 ± 0.2 in HG condition, *p* = 0.2995). To test whether the decrease in mEPSC frequency reflected a change in presynaptic vesicle release, we studied the paired-pulse ratio in response to two depolarizing stimuli delivered at 50 ms interval. The paired pulse ratio was not significantly different in NG and HG conditions (67.3 ± 4.9% [n = 10] and 61.7 ± 6.4% [n = 11], respectively, p = 0.5952), indicating that the HG-related depression of mEPSCs frequency was not due to changes in the initial release probability of presynaptic vesicles.

To extend our analysis to the postsynaptic side, we recorded the responses to glutamate-receptor activation by applying 100 μM glutamate through the extracellular solution perfusing the recorded neuron. In line with results on evoked EPSCs, the amplitude of the glutamatergic receptor-mediated current was not significantly different in autaptic neurons cultivated in NG or HG conditions (4.1 ± 0.4 nA vs 4.0 ± 0.5 nA, respectively, n = 14 each condition, *p* = 0.9910). Of note, with bath application of glutamate we stimulated the entire AMPA receptors pool expressed on cell membrane, instead of the synaptic pool only, without significantly affecting the NMDA receptors that, at resting membrane potential, are mostly blocked by extracellular Mg^2+^. To test whether glucose dyshomeostasis affected NMDA receptors, we measured evoked NMDA receptor-mediated currents by using a Mg^2+^-free external solution and suppressing the AMPA receptor-mediated component of the EPSC with 10 μM 2,3-dihydroxy-6-nitro-7-sulfamoyl-benzo(F)quinoxaline-2,3-dione (NBQX).

Under NG conditions, hippocampal autapses depolarized with stimuli mimicking action potentials evoked robust NMDA receptor-mediated currents (2.4 ± 0.4 nA). Conversely, HG significantly reduced the NMDA receptor-mediated currents (1.5 ± 0.3 nA, *p* = 0.03; Figures 4H–J). Interestingly, HG also significantly changed the decay time of NMDA receptor-mediated currents (426.8 ± 28.1 ms, in NG condition and 326.2 ± 38.0 ms, in HG condition, *p* = 0.04; Figures 4H,K). Finally, we measured the ratio between AMPA receptor-mediated and NMDA receptor-mediated EPSCs, which is a standard test to detect changes in synaptic strength (Kauer and Malenka, 2007). Autaptic hippocampal neurons grown in HG condition displayed a significant increase in the AMPA/NMDA ratio (3.9 ± 0.5, compared with 2.6 ± 0.4 seen in the NG autaptic hippocampal neurons; *p* = 0.0292, Figure 4L). Collectively, our data demonstrate that glucose overload impairs glutamatergic synaptic transmission at both pre- and post-synaptic levels.

## Discussion

Epidemiological evidence indicated that diabetic patients are significantly more susceptible to develop cognitive impairment, and elevated blood glucose levels increase the risk of dementia in both diabetic and non-diabetic individuals (Cukierman-Yaffee, 2009; Crane et al., 2013). Several molecular mechanisms have been proposed to underlie the hyperglycaemia-related alterations of brain plasticity, including the depletion of stem cell niche, the development of brain insulin resistance, microvascular complications and neuroinflammation (Hsu and Kanoski, 2014; Fusco et al., 2016; Spinelli et al., 2019). However, how glucose overload affects synaptic transmission and plasticity remains still poorly understood. Here, we found that a well-established animal model of hyperglycaemia, i.e., the STZ-injected mice, exhibited memory deficits (Figure 1B) associated with molecular changes in the hippocampus including lower amounts of NMDA receptor subunits GluN1 and GluN2a (Figure 1C), reduced phosphorylation levels of memory-related transcription factor CREB (Figure 1C) and decreased expression of genes encoding for synaptic proteins regulating synaptic transmission and plasticity such as SYT2, SYT4 and BDNF (Figure 1D). CREB is a pivotal hub in the activity-driven neuronal gene expression (Benito et al., 2011) and its activity has been reported to be critically reduced in the context of aging and age-associated brain diseases (Zuccato et al., 2001; Cui et al., 2006; Caccamo et al., 2010). In the last years, we identified CREB as novel metabolic sensor in the brain, whose transcriptional activity was finely regulated by the nutrient availability (Fusco et al., 2012; Fusco and Pani, 2013). To deeply investigate the CREB-related molecular and functional changes due to the glucose overload on hippocampal neurons, we studied the effect of medium containing HG levels on both hippocampal primary neurons and autaptic hippocampal neurons. Exposure of neurons to HG simulated the molecular changes observed in the hippocampus of STZ mice, including the inhibition of CREB activity (Figure 2B). More importantly, HG impaired the CREB phosphorylation induced by drugs mimicking neuronal activity and abolished the upregulation of synaptotagmins 2 and 4 (Figure 2B,C). This sort of “negative priming” of activity-dependent CREB response might be mediated by the inhibition of CREB activators AMP-activated protein kinase and Sirtuin 1 in high glucose condition (Fusco et al., 2012; Peng et al., 2016). Interestingly, it has been demonstrated that SYTs, in addition to control the neurotransmitter release on presynaptic side, can also play a critical role in regulating the exocytosis of postsynaptic receptors at postsynaptic level (Wu et al., 2017). Accordingly, SYT4 mutant mice showed deficits of hippocampus-dependent learning and memory (Ferguson et al., 2000).

Our electrophysiological experiments performed in autaptic hippocampal neurons indicated that glucose excess impaired the spontaneous release of glutamate from presynaptic terminals whereas the evoked release and paired-pulse ratio were not affected (Figure 4). The decreased mEPSCs frequency we observed could be attributed to the downregulated expression of key proteins involved in synaptic vesicles fusion (Figure 2). However, other mechanisms might be also involved. First, glycaemia homeostasis imbalance has been reported to decrease intracellular Ca^2+^ levels (Chan and Greenberg, 1991). Another possible explanation of mEPSCs frequency alteration observed in HG-treated neurons is that the number of vesicles in presynaptic terminals and in the readily releasable pool of synaptic vesicles were different in NG- and HG-treated neurons. Furthermore, prolonged exposure to high levels of extracellular glucose may induce insulin resistance desensitizing insulin receptors. Specifically, it has been demonstrated that downregulation of insulin receptors signaling resulted in a significant reduction in the frequency of mEPSCs without affecting either the distribution of their amplitudes or the presynaptic release probability (Chiu et al., 2008; Lee et al., 2011).

To answer the fundamental question on how HG adversely impacts synapse function, we extended our analysis to the postsynaptic site by recording the NMDA receptor-mediated currents. We observed that HG differentially affected the evoked AMPA and NMDA receptor-mediated currents. Specifically, AMPA receptor-mediated currents were unaffected by HG treatment (Figures 4B,C). Conversely, we observed a significant reduction of NMDA receptor-mediated currents together with a reduction of the decay time in HG-treated neurons (Figure 4H–K). These data, including the increased AMPA/NMDA ratio observed in HG neurons, suggest that glucose dyshomeostasis preferentially targets NMDA receptors, although our Western blotting analysis performed in the hippocampus of STZ mice revealed a significant reduction of GluA2 subunits (Figure 1C). Of note, the streptozotocin-induced type 1 diabetes model is characterized by more complex metabolic changes, including drastic decrease of insulin levels and alteration of leptin signalling, which may explain the differences between our *in vivo* and *in vitro* models (MacDougald et al., 1995). NMDA is a tetrameric receptor with two obligatory GluN1 subunits and two regulatory subunits, GluN2A and GluN2B (Yashiro and Philpot, 2008). The kinetics of NMDA receptor mediated currents reflect a different subunit composition of NMDA receptors which influences their Ca^2+^ permeability. Faster kinetics indicates lower Ca^2+^ influx through NMDA receptors (Yashiro and Philpot, 2008; Lee et al., 2010). In HG neurons, we found faster NMDA decay times suggesting lower Ca^2+^ influx (Figures 4H,J). Thus, we are proposing that glucose excess would influence the threshold for synaptic plasticity by affecting synaptic metaplasticity. Intriguingly, spontaneous glutamate release, instead of evoked release, adjusts functional and structural plasticity threshold at single synapses by local regulation of NMDA receptors (Lee et al., 2010). Our data support the idea that dietary regimen may influence brain plasticity, at least in part, by modifying CREB activity via altered glucose metabolism homeostasis (Mainardi et al., 2012). Of note, the beneficial effects of calorie restriction on synaptic plasticity and memory were abolished in mice lacking CREB in the forebrain (Fusco et al., 2012). However, glucose excess could also negatively impact on synaptic function by changing the intracytoplasmatic Ca^2+^ clearance (Nakashima et al., 1996), enhancing oxidative stress (Treviño et al., 2015) and impairing astrocyte energy metabolism (Li et al., 2018).

Here, we identified novel CRE regions on the regulatory sequences of SYT 2 and SYT4 genes, which may trigger the HG-dependent changes of synaptic function (Figure 3A). A fundamental question is whether SYTs also contribute to postsynaptic responses during neurotransmission. SYT4 deficiency has been demonstrated to modify the release of neurotrophic factor BDNF at postsynaptic level (Dean et al., 2009). BDNF has been recognized as strong modulator of multiple neuronal functions including synaptic plasticity, learning and memory (Minichiello, 2009; Fusco et al., 2019). Collectively, our data provide new insights into the glucose-responsive CREB modulation of synaptic proteins regulating synaptic vesicle release. An intriguing hypothesis is that glucose availability influences the activity-dependent recruitment of CREB to the synaptotagmin promoters (Figure 3B) and the level of synaptic proteins controlling the vesicle release. The inhibition of spontaneous glutamate release together with the decrease of neurotrophin levels could contribute to synaptic function deficit observed in experimental models of diabetes. Specifically, the observed defects in mEPSC frequency may adversely affect the activity of NMDA receptors, which in turn regulate synaptic plasticity, learning and memory. Moreover, lower expression of synaptic proteins may elicit the decrease of both dendritic branching and spine density observed in experimental models of hyperglycaemia (Malone et al., 2008).

As mentioned above, hyperglicaemia and alteration of glucose homeostasis have been implicated in age-dependent cognitive decline and memory loss, although the molecular mechanisms are still elusive. Our findings reveal a novel molecular circuit that regulates synaptic transmission at pre- and post-synaptic levels involving CREB-dependent-downregulation of SYTs. A different model of insulin resistance-dependent hyperglycaemia, i.e., high fat diet (HFD)-fed mice, showed similar memory deficits and impairment of synaptic functions compared to STZ mice that were primarily attributed to aberrant protein palmitoylation (Yan et al., 2016; Spinelli et al., 2017). HFD also inhibits CREB phosphorylation in the hippocampus (Wu et al., 2018), as well as the expression of genes encoding SYT2 and SYT4 in mouse cerebral cortex (Yoon et al., 2019). However, despite sharing several functional and behavioral alterations, HFD and STZ models also differ for a number of intracellular molecular cascades relying on insulin resistance primarily occurring in the former. Future studies are needed to better understand the role of glucose-driven CREB transcriptional activity in age-dependent memory loss and its potential impact on personalized medicine approaches.

## Data Availability Statement

The raw data supporting the conclusions of this article will be made available by the authors, without undue reservation.

## Ethics Statement

The animal study was reviewed and approved by the Ethics Committee of Università Cattolica del Sacro Cuore and were fully compliant with Italian (Ministry of Health guidelines, Legislative Decree No. 116/1992) and European Union (Directive No. 86/609/EEC) legislations on animal research.

## Author Contributions

CR, SF, and CG conceived the study, supervised the work, and wrote the manuscript. CR performed the electrophysiological experiments. MS performed the metabolic analyses and western blotting experiments. FN performed the gene expression analysis. SF designed and performed the behavioral and ChIP experiments. All authors commented on the manuscript and approved its final version.

## Conflict of Interest

The authors declare that the research was conducted in the absence of any commercial or financial relationships that could be construed as a potential conflict of interest.
