# Supplementary material for: Glucose Overload Inhibits Glutamatergic Synaptic Transmission: A Novel Role for CREB-Mediated Regulation of Synaptotagmins 2 and 4
Source: Front Cell Dev Biol. 2020 Aug 19;8:810. doi: 10.3389/fcell.2020.00810 (PMC7466440; doi:10.3389/fcell.2020.00810)
Supplement: Supplementary file 1 [file Table_1.DOCX]

**Supplementary Table 1. ANTIBODIES**

| **Primary Antibody** | **Host** | **Catalogue reference** |
| --- | --- | --- |
| α-GluA1 (N-terminus) | Mouse | Millipore #2263 |
| α- GluA2 | Mouse | Millipore #391 |
| α-GluN1 | Mouse | BD Bioscience #556308 |
| α-Actin | Rabbit | Biorbyt #10033 |
| α-GluN2A | Mouse | Santa Cruz #515148 |
| α-GluN2B | Mouse | BD Bioscience #610417 |
| α-CamKII | Mouse | Santa Cruz Sc-32288 |
| α-pCamKII^T286^ | Rabbit | Cell Signaling 12716 |
| α-pCreb^S133^ | Rabbit | Millipore 06-519 |
| α-Creb | Rabbit | Millipore 06-863 |
| α-Syt4 | Mouse | Santa Cruz Sc-271936 |
| α-MAP2 | Mouse | Sigma Aldrich M9942 |
