# Supplementary material for: Glucose Overload Inhibits Glutamatergic Synaptic Transmission: A Novel Role for CREB-Mediated Regulation of Synaptotagmins 2 and 4
Source: Front Cell Dev Biol. 2020 Aug 19;8:810. doi: 10.3389/fcell.2020.00810 (PMC7466440; doi:10.3389/fcell.2020.00810)
Supplement: Supplementary file 2 [file Table_2.DOCX]

**Supplementary Table 2. PRIMERS**

Primer sequences used for Real Time PCR analyses.

| **Gene** | **Primer sequence** | |
| --- | --- | --- |
| *Syt1* | FW | 5’-CGATTAAGAAGAACACACTTAACC-3’ |
|  | RV | 5’-CTCTACCTGCAGAGTGTGCC-3’ |
| *Syt2* | FW | 5’-GACGACAGTGAAGAAGAAGACC-3’ |
|  | RV | 5’-TCGGTGCCTGTGGCGTTGC-3’ |
| *Syt4* | FW | 5’-TACCGAAATCTGATGTGTCTGG-3’ |
|  | RV | 5’-GATTTCTTCAAGACTCTCACAAGG-3’ |
| *Syp* | FW | 5’-CTTCTCCATCAGATGTAATCTGG-3’ |
|  | RV | 5’-CTGAGGAGTGCAAGGTCAGG-3’ |
| *Syn1* | FW | 5’-TGGTCCAGCTCCAACGAAGG-3’ |
|  | RV | 5’-GTCTCAGCTTTCACCTCGTCC-3’ |
| *Bdnf* | FW | 5’-TGGCTGACACTTTTGAGCACG-3’ |
|  | RV | 5’-GAAGTGTACAAGTCCGCGTCC-3’ |
| *Gapdh* | FW | 5’-TCCATGACAACTTTGGCATT-3’ |
|  | RV | 5’-GTTGCTGTTGAAGTCGCAGG-3’ |

Abbreviations: FW, forward; RV, reverse; Syt, synaptotagmin; Syp, synaptophysin; Syn, synapsin.

Primer sequences used for ChIP analyses.

| **Gene** | **Primer sequence** | |
| --- | --- | --- |
| SYT2  promoter | FW | 5’-AGCGGTTGTGGAGGTCAGG-3’ |
|  | RV | 5’- CGTGCCTGCGGGACTGTCC-3’ |
| SYT4  promoter | FW | 5’-TGGTGAGTGTGCGCTGAATCC-3’ |
|  | RV | 5’-CGCTCTCTATCAGCAATGTGC-3’ |
